# Supplementary figures and images for: Adherence to the Mediterranean Diet Is Associated with a More Favorable Left Ventricular Geometry in Patients with End-Stage Kidney Disease
Source: J Clin Med. 2022 Sep 28;11(19):5746. doi: 10.3390/jcm11195746 (PMC9571193; doi:10.3390/jcm11195746)

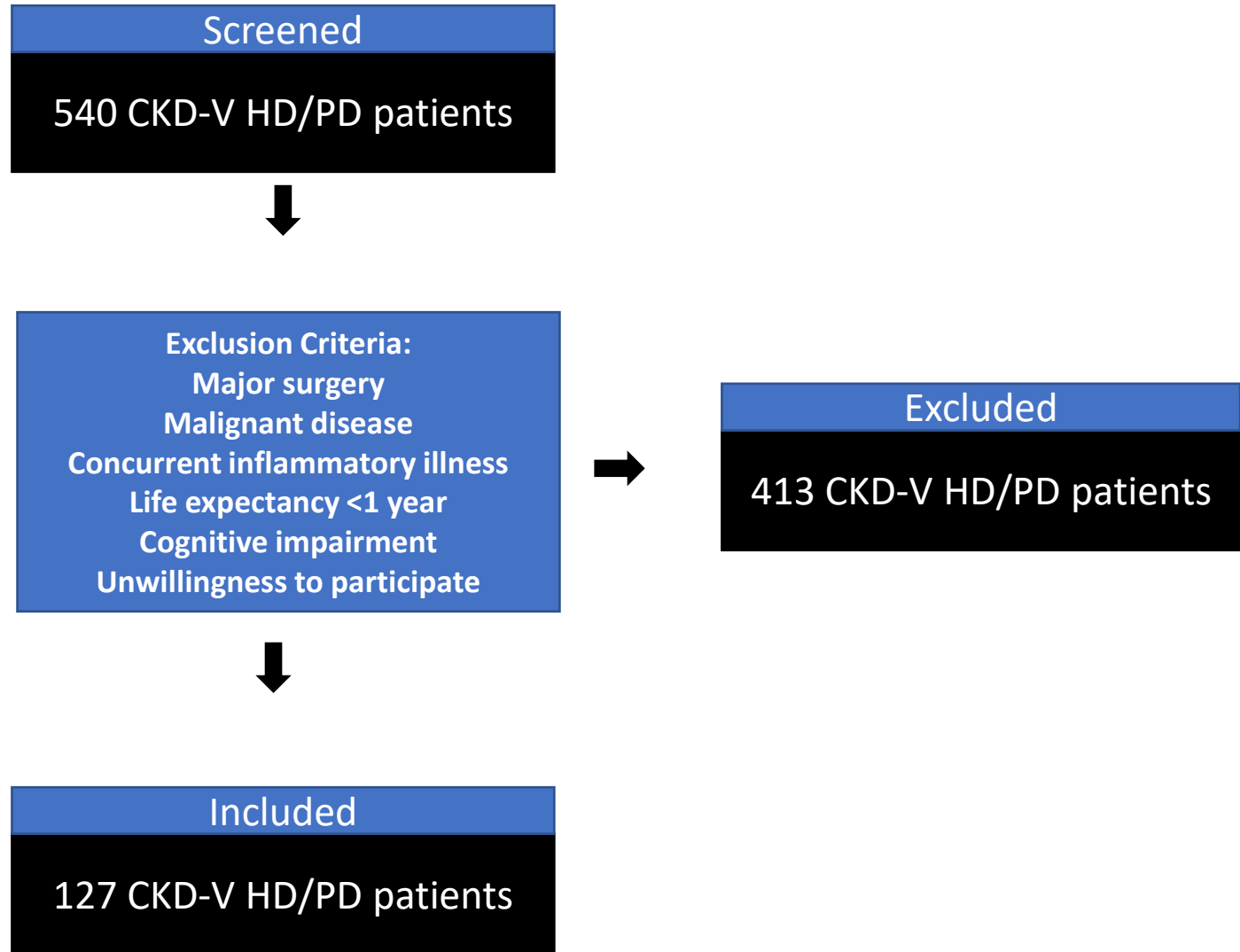

Figure S1. Flow diagram of patients recruited into the study.

Supplement: Supplementary file 1 [file jcm-11-05746-s001.zip › jcm-1887070-supplementary2.pdf]
